# Ethnobotanical contributions to global fishing communities: a review

**DOI:** 10.1186/s13002-023-00630-3

**Published:** 2023-12-02

**Authors:** Jimlea Nadezhda Mendoza, Natalia Hanazaki, Baiba Prūse, Agnese Martini, Maria Viktoria Bittner, Sophia Kochalski, Edison Macusi, Aimee Ciriaco, Giulia Mattalia, Renata Sõukand

**Affiliations:** 1 Department of Environmental Sciences, Informatics and Statistics, Ca’ Foscari University of Venice, Venice, Italy; 2 Tagalog Fisher Community of Mabato Asufre Pangil, Pangil, Laguna Philippines; 3 Department of Ecology and Zoology, Federal University of Santa Catarina, Florianópolis, Brazil; 4 Athena Institute, Vrije Universiteit Amsterdam, Amsterdam, The Netherlands; 5 Department of Asian and North African Studies, Ca Foscari University of Venice, Venice, Italy; 6 CRETUS, Department of Applied Economics, University of Santiago de Compostela, Santiago de Compostela, Spain; 7 Faculty of Agriculture and Life Sciences, Davao Oriental State University, Mati, Philippines; 8 Kabulusan Integrated National High School, Pakil, Laguna Philippines; 9 Institut de Ciència i Tecnologia Ambientals, Universitat Autònoma de Barcelona (ICTA-UAB), Cerdanyola del Vallès, Barcelona, Spain; 10 New York Botanical Garden, New York, USA

**Keywords:** Local ecological knowledge, Fisherfolk, Traditional ecological knowledge, Traditional fisheries knowledge, Ethnobiology, Plant uses, Traditional plant knowledge, Indigenous plant knowledge

## Abstract

**Background:**

Ethnobotanical knowledge about the role of plants in fisheries provides valuable ecological information vital for sustainable management of local resources; however, it is diluted and understudied globally. This literature review aims to map the knowledge of plant use within traditional fishing communities.

**Methods:**

Through the PRISMA method, we identified and selected 34 articles reporting the use of plants in fisheries, and including 344 taxa of plants and algae. Uses of plants and algae were grouped into different categories.

**Results:**

In the novel categorization of fishery-related uses we proposed, the most mentioned were for fishing and building/repair of fishing artifacts and habitat-related uses, while the records of plants related to fiber uses, providing aid in fishing management and species causing problems, were among the least mentioned. Semi-structured interview is most commonly used with local resource users, especially fishery experts, in exploring perceptions on plant use within traditional fishing communities. Diversity was high in all the recorded families, but most were reported locally.

**Conclusion:**

Ethnobotanical studies with fishers are not common in the documented literature but they provide a large number of use reports. On the basis this review, in most of the world, the information is of a casual and sporadic nature. Fishers can provide information on aquatic plants and algae that create problems and aid in fishing management, which are crucial in understanding the ecosystem of a region experiencing environmental challenges. This knowledge is greatly understudied globally and undergoing a rapid decline, as highlighted in several of the reviewed articles. Thus, further systematic research on fishery-related uses of plants by fisherfolk is needed considering its potential contribution to the sustainable management of fishery resources.

**Supplementary Information:**

The online version contains supplementary material available at 10.1186/s13002-023-00630-3.

## Introduction

Artisanal fisheries are widespread across the globe, with regional particularities and differences, and the diversity and use of natural resources related to this activity usually depends on their local availability. Traditional fishery knowledge, as other forms of traditional ecological knowledge (TEK), has evolved over the millennia, and is based “on the use of natural materials for construction of tools, vessels and equipment,” as well as the “observations of weather patterns, sea conditions, and the accumulation and transmission of that knowledge about fishing and fishing related activities [1].” To enable the plants suitable for specific uses or for specific environments, the choice of plants used in fisheries is based on its properties and availability [1], and these uses and choices are mediated by traditional ecological knowledge of fishers. Traditional ecological knowledge (TEK) is passed through generations through cultural transmission and serves as a crucial response to the changes occurring in the environment as TEK is a major source of community cohesion and resiliency [2, 3].

The field of study known as ethnobiology, among others, including the in-depth traditional or local ecological knowledge (LEK), is typically maintained by local human communities who have long used and managed natural ecosystems [4–6]. Silvano et al. [7] highlighted the potential contributions of ethnobiology to other research areas by reporting case studies that will improve ecological research and further engage local communities in protecting forest-stream ecosystems. For example, riverine people in the Amazon have broad perceptions providing alternative views on the humans and the environment relations, contributing novel observations that complement existing knowledge, such as the information about fish populations and biotic/abiotic variables affecting their development [8]. Bhatta and Patra [9] reported that the knowledge shared by local people is crucial in understanding an ecosystem, as it could contribute to conserving threatened native wetland species. In numerous study fields, including fish ecology and fisheries, studies about TEK and LEK have provided new biological information, as well as contributed to the development of management and conservation measures [10–16], including the restoration of damaged landscapes due to agricultural activities in rural landscapes in Australia [17]. Several investigations, especially in the coastal region of the Brazilian Atlantic Forest, have documented knowledge of and usage of plants by fisherfolk [18–24], and fishing ecology [19], among others. Traditional ecological knowledge and LEK are also related to climate and environmental changes [25] and watershed rehabilitation [26]. For example, Sethi et al. [27] claimed that climate change-related challenges and variability could be solved using traditional indigenous knowledge, as the local people observing these issues are among the first ones to adapt to them because they depend on biodiversity for their livelihood. However, many practices concerning plant species disappeared from daily activities, especially those related to traditional fishing [28] For instance, a decrease in traditional ethnobotanical fishery knowledge has been reported along the Western Mediterranean Italian coast and on its small islands due to environmental issues such as climate change (seasonality), the decline of fish stocks, and also tourism activities [1, 29].

Tng et al. [30] argued that the knowledge of traditional experts on plant use is undergoing a progressive dilution from one generation to the next, claiming that further studies on the succeeding generation’s knowledge of plant use are necessary due to its decline. Research on the knowledge, usage, and management of natural resources by local populations is crucial because it validates the value of cultures and advances the ability of the community to sustain itself [31]. Hanazaki et al. [32] investigated that the interrelation among fishing, people, and plants in coastal environments has rarely been the topic of ethnobotanical studies and fishing activities about plants and their role in ethnobotanical knowledge are not very well documented. This is shown by the relatively data obtained from bibliographic sources [1]. Therefore, documenting this traditional ecological knowledge before it disappears from oral history is crucial in decreasing the loss of TEK and biocultural diversity [3]. Several studies demonstrated that the preservation of this local knowledge (and social memory) is vital for sustainable management of the environment and for dealing with socioecological changes [33, 34].

In addition to the basic biological and ecological perspective, investigations into biodiversity and conservation efforts should include concerns about the use of biodiversity in their equations. As a multidisciplinary field, ethnobotany offers a variety of methods and viewpoints to encourage communication between various areas of knowledge [35].

Therefore, this review aims to understand the general scenario of fishery-related plant uses in the published literature worldwide. To this end, we mapped plant use knowledge within traditional fishing communities across the globe and reviewed the methods used in investigating the plant use knowledge within traditional fishing communities across the globe. We also identified the respondents commonly involved in exploring the plant use knowledge within traditional fishing communities across the globe and provide the inventory of the reported plants utilized in fisheries and their corresponding uses.

## Methodology

### Literature review

**Fig. 1:**
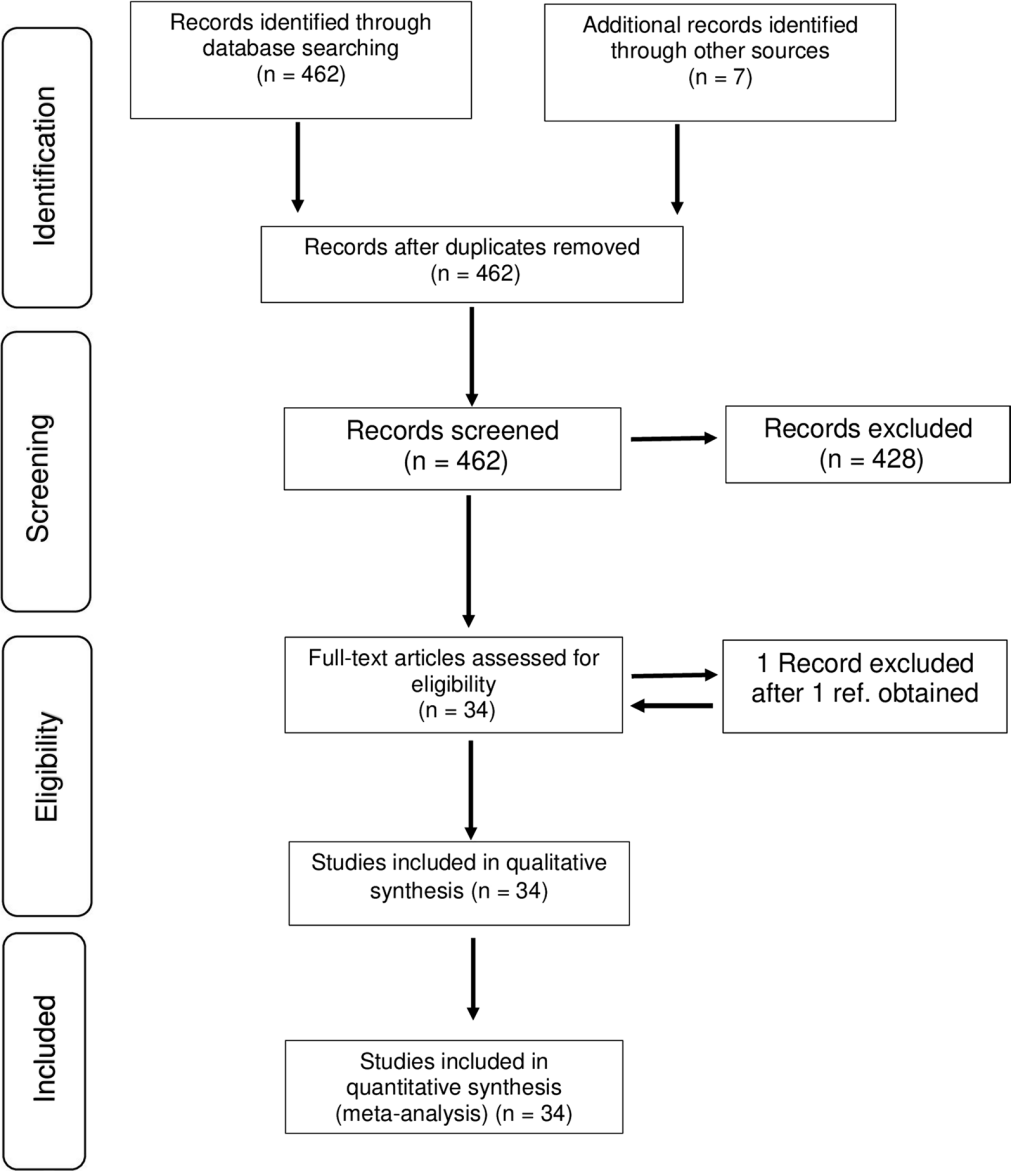
Flow diagram for the literature review with the Preferred Reporting Items for Systematic Reviews and Meta-Analyses (PRISMA)

For the systematic literature review, we used the Preferred Reporting Items for Systematic reviews and Meta-analyses (PRISMA) to guide the review process [36] (Fig. 1).

We searched for published literature in English using the following key words in Scopus database: “knowledge” AND “use” AND “fisheries” AND “management.” Only the articles or reviews involving local perceptions about the uses of plants were considered because they have undergone a peer review process.

Given that the results of the initial search did not yield sufficient fishery-related uses of plants based on local ecological knowledge, a second search was similarly performed using the terms “fisher” OR “fishery” AND “plants” AND “knowledge” OR “use” AND “local” OR “indigenous” OR “traditional.” Furthermore, an additional search was performed in the Web of Science database using the keywords “fishers’ knowledge,” “plant,” and “use.” All searches included articles published between 1970 and 2023.

Additional peer-reviewed articles were also considered for being part of the references that did appear in the searches. A study was selected only if it followed the above-mentioned criteria or if it involved local perceptions about the uses of plants related to fisheries. These studies were considered as they were in line with the research aims. Excel databases were created showing the use reports (see Additional file 1) and all the articles considered in the review (see Additional files 2 and 3), including some background information about the articles such as the methods and respondents involved.

Initially, articles were screened by their titles and abstracts, which had to report the perception and uses of plants by resource users in the region for their fishing activities. Fishery-related uses of plants were obtained from studies where the authors used different terminologies such as traditional fishery knowledge, traditional ecological knowledge, local ecological knowledge. In the cases where medicinal, housing, and construction, fodder, among other uses, were reported, these were not considered in this review. After screening abstracts, full texts were analyzed to determine if they were eligible for the final synthesis. The eligibility criterion was that publications had to report fishery-related uses of plant species based on LEK.

The number of publications decreased from 113 to 9 after screening using the first set of terms in Scopus. For the results of the second search, the number of publications decreased from 123 to 21 articles after the screening process; however, seven articles out of 21 appeared in the results of the previous selection process, and thus, only 14 articles reporting only fishery-related uses were added in the final review. Furthermore, for the results of the Web of Science database search, the number of publications decreased from 226 to 10 including [37, 38]; however, 5 articles also appeared in the results of the Scopus search. Therefore, 28 articles were finally included in the final review process. Six additional peer-reviewed articles were added, as those also reported fishery-related uses of plant species based on LEK. They did appear while exploring the reviewed articles but were not listed in the results of the Scopus and Web of Science searches. The final analysis was performed on 34 publications (Fig. 1).

#### Data categorization

Uses were grouped into larger categories including aids in fishery management, building and repair, fiber uses, fishing, habitat, and problems (Table 1). From each article, we extracted the uses of plants related to fisheries and we grouped them into these larger categories.

**Table 1:** General categories of use

General categories	Use	Abbreviation
Aids in fishing management	Aids in fishery management	AIF
	Check pH	CPH
	Control algal bloom	CAB
	Control disease in fish	CDF
	Control of humus gas	CHG
	Control of snails	COS
	Control of unwanted fishes	CUF
	Control of wild cat	CWC
	Faster in hatching	FIH
	Fish conservation	FCS
	Mapping of fish resources	MFR
	Prevent mortality in Transportation	PMT
	Reduction of water turbidity	RWT
	Seasonal cues for fish presence	SCF
	Signify octopus season	SOS
	Signify sharks giving birth	SGV
	Site for catching fish	SCF
	Water filter	WAF
Building and repair	Barrels	BAR
	Build boats	BOT
	Build canoes	CAN
	Build ships	SHP
	Build temporary fishing camps	BTF
	Caulking	CAU
	Coloring	CLR
	Construct boat shelters	CBS
	Cover boats	CVB
	Dye nets	DYN
	Fishing net floats	FNF
	Floats	FLT
	Fuel for cooking fish	FCF
	Grilling	GRL
	Hulling	HUL
	Make oars	OAR
	Make ships go faster	SGF
	Masts	MAS
	Paddles	PAD
	Pulleys	PUL
	Ramps	RAM
	Repair canoes	RCA
	Repair fishing nets	RFI
	Ship models	SHM
	Shrouds	SHR
	Splash battress	SPB
	Tools	TOL
Fiber uses	Basketry	BAS
	Broom heads	BHE
	Fish nets	FSN
	Ropes	ROP
	Use as cordage in fishing activities	COR
	Weaving fish traps	WFT
Fishing	Bait	BAI
	Catching fish	CAF
	Fish poison	FPO
	Fish traps	FST
	Fishing	FIS
	Fishing gear	FSG
	Food for fish	FFF
	For harvesting fish	FHF
	Hooks	HOO
	Illegal fishing	ILF
	Making fish traps	MFT
	Making fishing rod	FRD
	Mussel farming	MSF
	To get octopus out of its den	TOD
	To stun fishes	TSF
Habitat	Dams of fish ponds	DFP
	Fish habitat	FSH
	Helps lower the cost of fish feeds	LCF
	Hiding place	FHP
	Protects the fish from predators	PFP
	Provides shade	SHD
	Stabilize soil	STS
	Stabilize temperature	STT
Problems	Food for people (overexploitation)	FOP
	Invasive species causing problems in fishing	ICI
	Problem contributing to fish kill	PCF
	Problem for fishing activities transportation	PFT
	Problem for recreational fishing	PRF
	Source of income (overexploitation)	SOI

The categories were based on the following criteria. Fishing-related uses are grouped together when they are related specifically to catching fish. Building and repair-related uses involve the plant materials for building, constructing, and repairing fishing gear. Fiber-related uses included making use of the plants’ fiber. Habitat-related uses refer to the plants’ function as habitat for the fish and other aquatic organisms. Problem-related uses represent the challenges plants bring to fishing activities, for instance, due to overgrowth. Lastly, aids in fishery management-related uses reflect on the contribution of plants in fishery management.

Botanical names were standardized and listed following the Plants of the World Online database [39].

The data on plant uses and botanical identifications were organized in spreadsheets, followed by pivoting techniques to obtain a summary of the results, especially those concerning plant use reports related to fisheries mentioned in the reviewed articles. Comma-separated values (CSV) files were generated for data visualization in Rawgraphs [40].

## Results

**Fig. 2:**
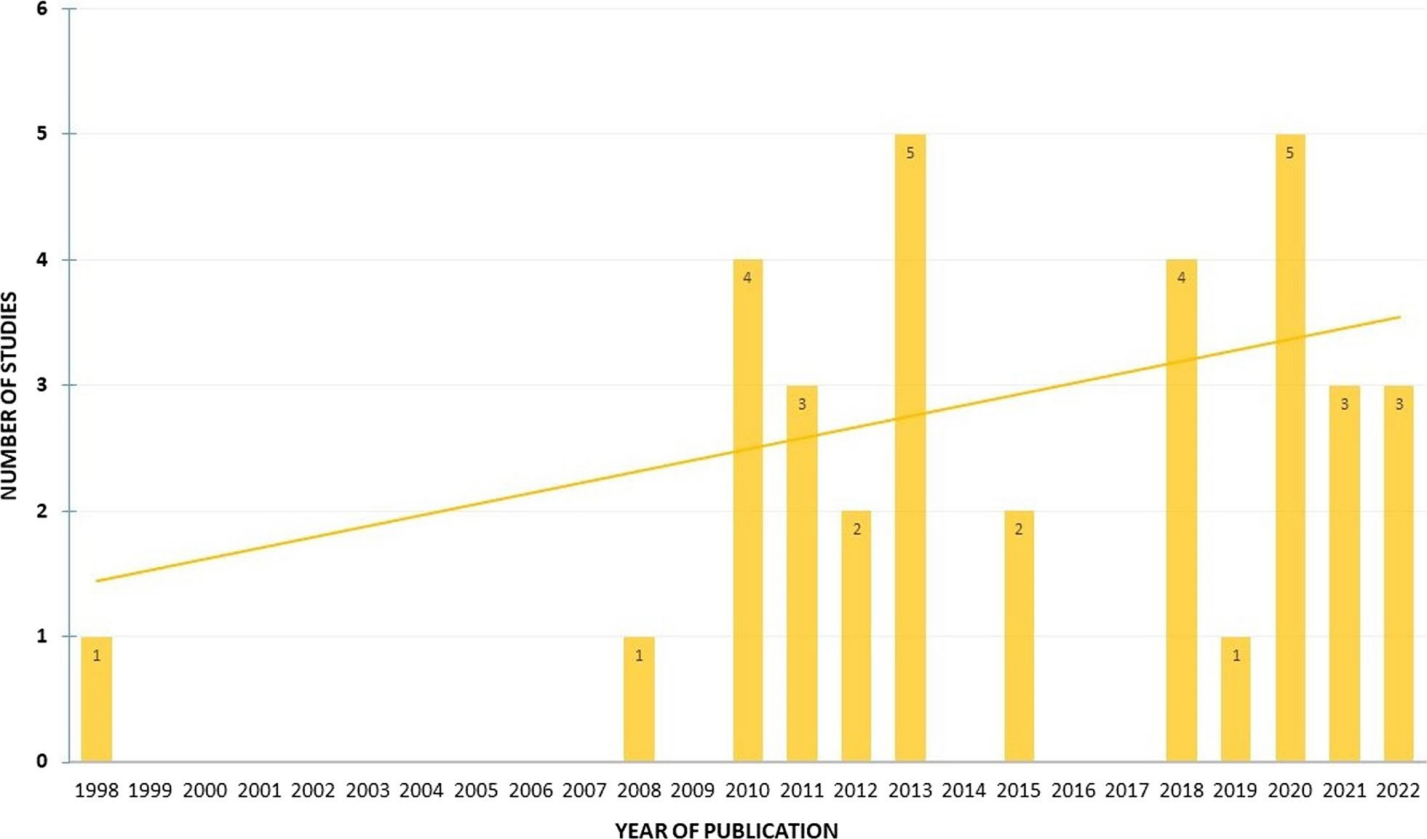
Summary of the publication year of the studies included in the review

**Fig. 3:**
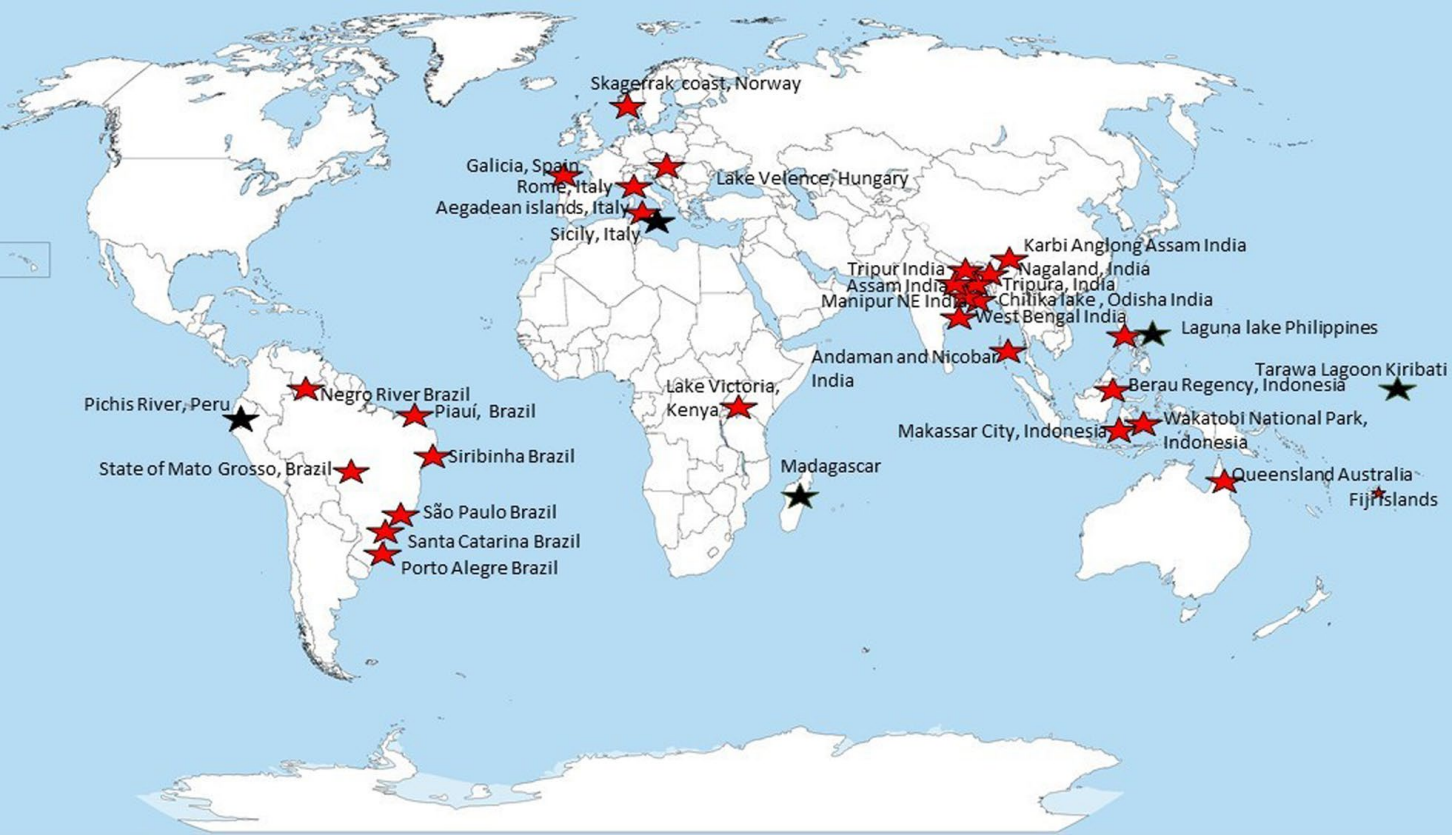
Distribution of the reviewed articles on the world map, red stars mean original reasearch, black stars mean original research combined with other data sources Source of the base map: https://upload.wikimedia.org/wikipedia/commons/c/cf/A_large_blank_world_map_with_oceans_marked_in_blue.PNG

The earliest article corresponding to the selected criteria was published in 1998. There has been a slight increase in recent years in the number of published articles on fishery-related plant uses based on the knowledge of local people (Fig. 2). South America is the most researched region with 286 use reports followed by Europe (203 use reports), while among the least explored continents were Asia (87), Australia (44), and Africa (14) (Fig. 3).

Most of the publications are original research papers (29 out of 34) in which the information is only based on local ecological knowledge, while the five publications combine original research with other methods such as a literature review (indicated by black stars in Fig. 3). The authors of the reviewed articles refer to the source of fishery-related plant and algae uses as traditional fishery knowledge, traditional ecological knowledge, traditional botanical knowledge, local ecological knowledge, and indigenous technical knowledge.

**Fig. 4:**
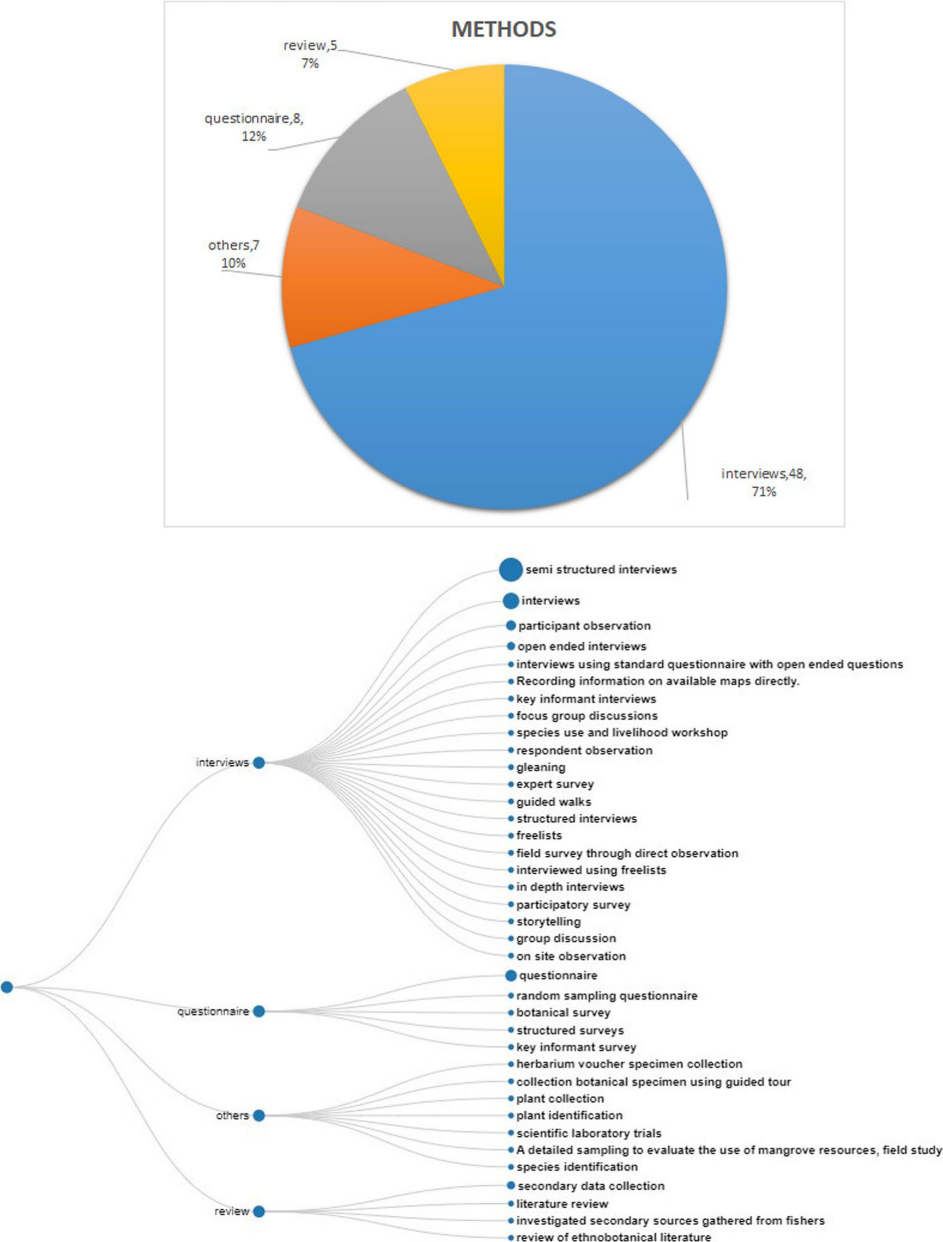
The methods of data acquisition reported in the included articles

Most of the reviewed publications are based on (semi-structured) interviews (Fig. 4). Botanical surveys, random sampling questionnaires and structured surveys are less well represented. Nine percent of the publications also rely on secondary sources including those collected from fishers, available records from government agencies such as fisheries, fishers focus group discussions, and reviews of ethnobotanical literature.

**Fig. 5:**
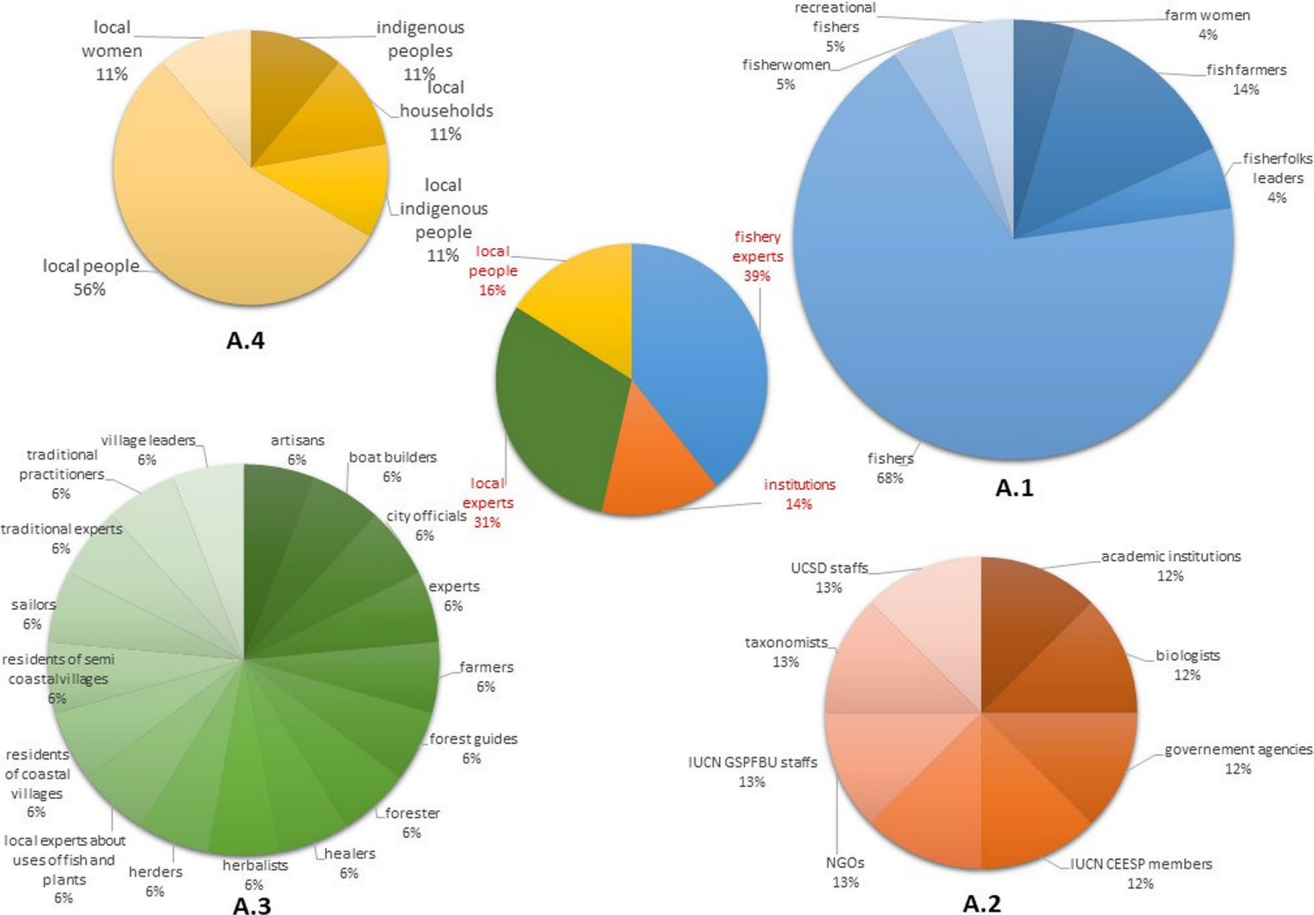
Knowledge sources reported in reviewed articles

**Fig. 6:**
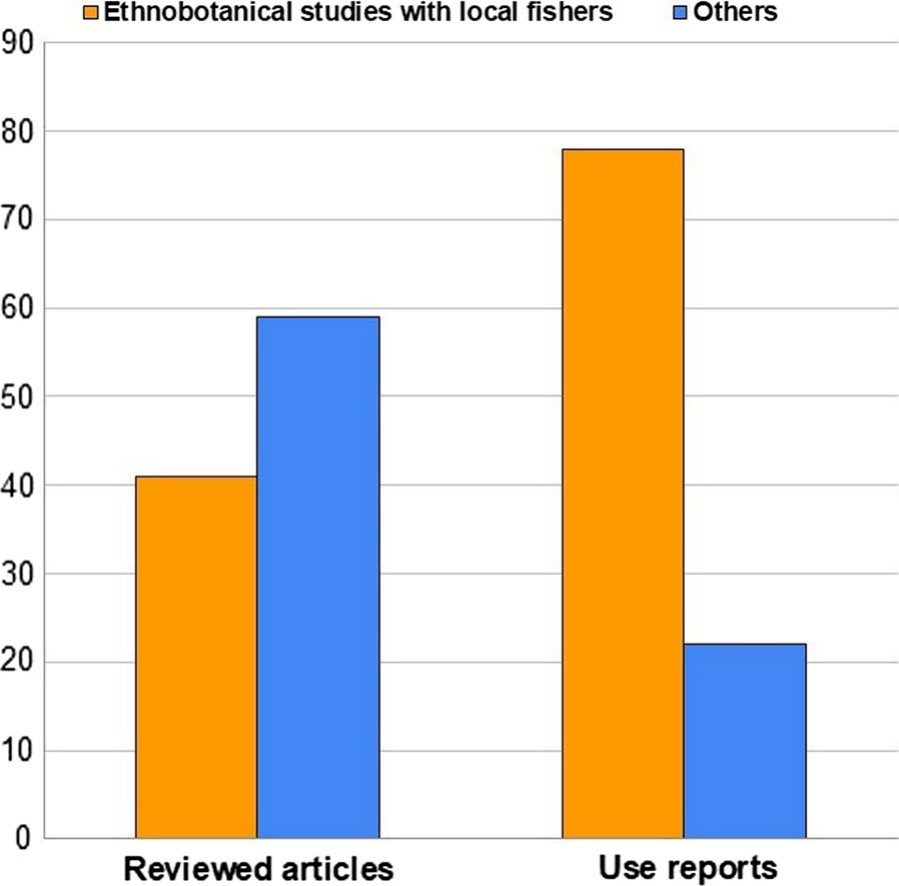
Percentage of use reports from ethnobotanical studies with local fishers globally

The respondents (Fig. 5) in the publications involved in determining the fishery-related uses of plants are mostly fishery experts and local experts followed by other local people and institutions. The majority of fishery experts are fishers, while local experts represent people from very diverse professions, including fishery-related artisans among others. Globally, the majority (78%) of the use reports derive from records obtained from ethnobotanical studies involving fishery experts, which comprise less than half (41%) of the reviewed articles (Fig. 6). The institutions involved are academic institutes, biologists, government agencies, and members of organizations such as IUCN CEESP, IUCN GSPFBU, UCSD, and various NGOs.

A total of 344 plant and algae taxa belonging to 112 botanical families were identified. In ten records, only common name was mentioned, 70 plants were reported at genus level and two records provided plant descriptions (see Additional File 1). Eight records had unidentified families; therefore, only 556 records of use reports were identified to the level of species. Among the most cited plant taxa used were *Castanea sativa* and *Pontederia crassipes* (10 use reports each), followed by *Ampelodesmos mauritanicus, Arundo donax, Bambusa sp., Nectandra,* and *Ocotea* (8 use reports each), *Hymenachne amplexicaulis, Montrichardia linifera*, *Myrsine guianensis*, *Myrtus communis, Olea europaea, Rhynchospora corymbosa*, and *Rugoloa polygonata* (6 use reports each).

**Fig. 7:**
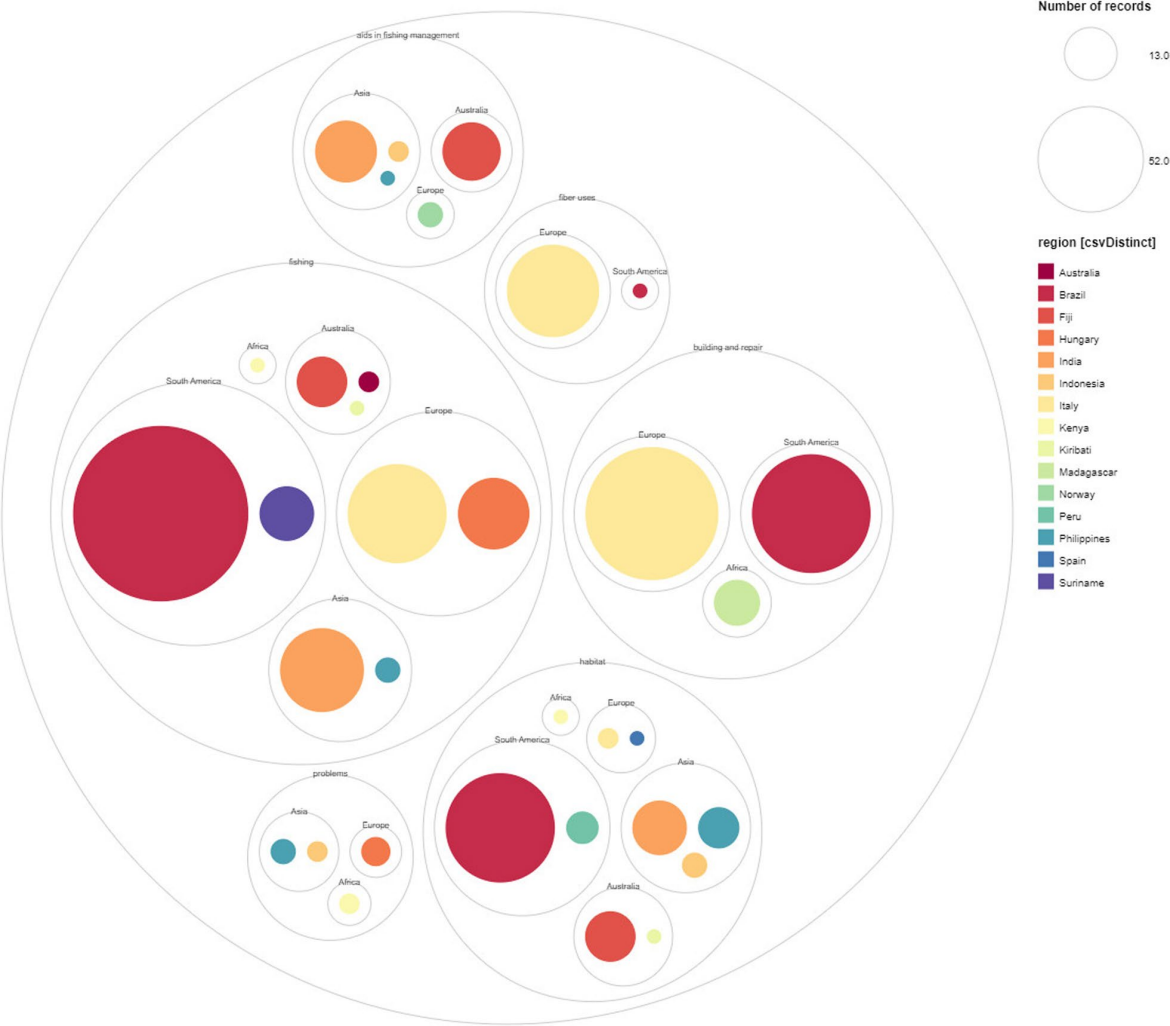
Frequency of reports on fishery-related uses of plants in reviewed articles per geographical area

The fishery-related uses of plants are not region-dependent (Fig. 7). However, they reflect the research effort in each specific region. Specifically, publications in Brazil showed the highest number of reported plant uses (276 out of 634) related to fishery, followed by publications in Italy (171) and India (65). The numbers correspond to the number of publications: Most studies (*n* = 8: 23.53%) were carried out in Brazil and India, followed by Italy and Indonesia (*n* = 3: 8.82%), while in other reported regions only one or two studies have been conducted. Most of the diversity also comes from Brazil, where the majority of the plant families are used in a local context.

**Fig. 8:**
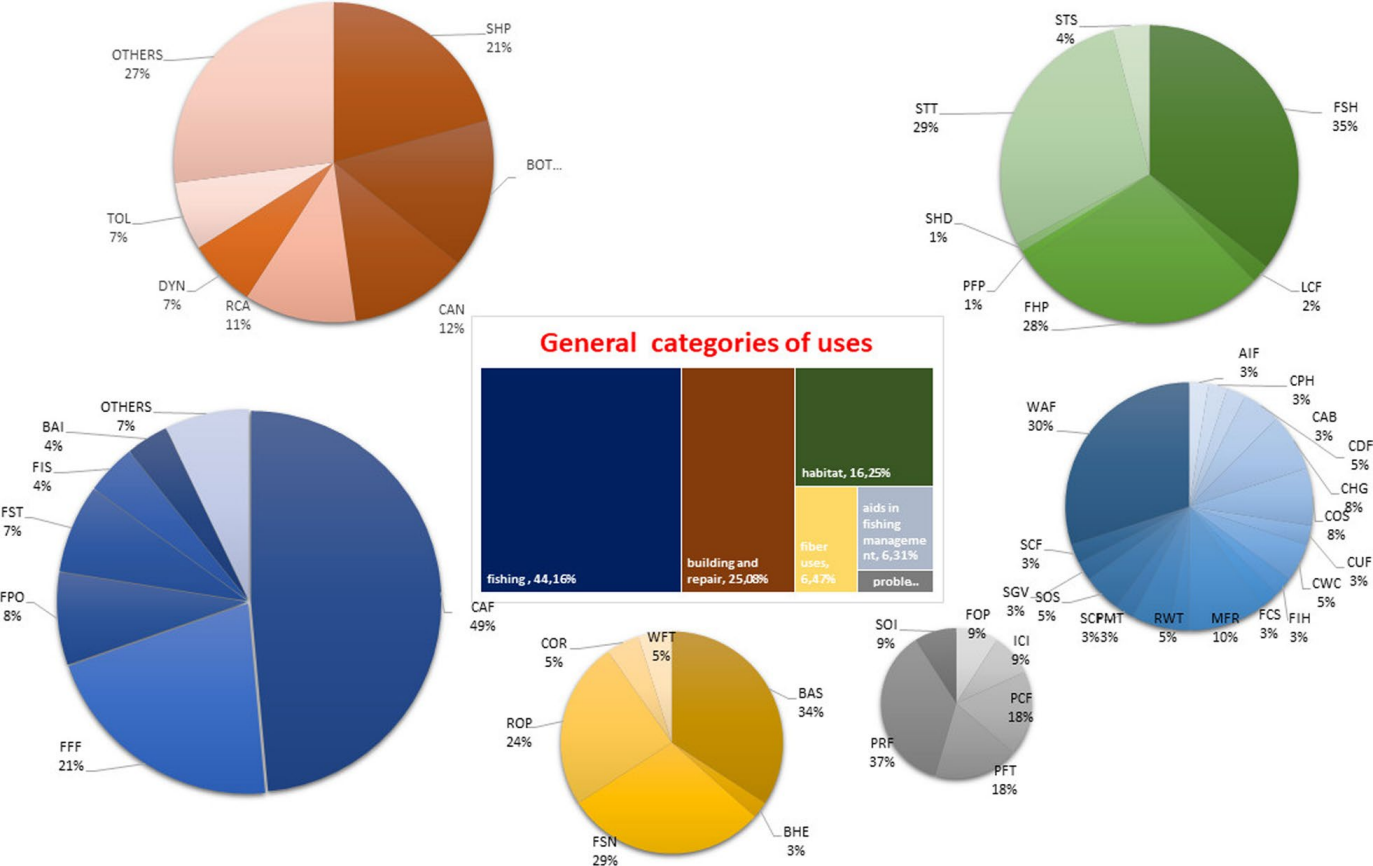
General categories of fishery-related uses of plants in the world

Uses were grouped into general categories, such as fishing (44.16%), building and repair (25.07%), habitat (16.25%), and fiber uses (6.47%) and aids in fishing management (6.31%); records concerning plant species causing problems were the least mentioned (Fig. 8).

**Fig. 9:**
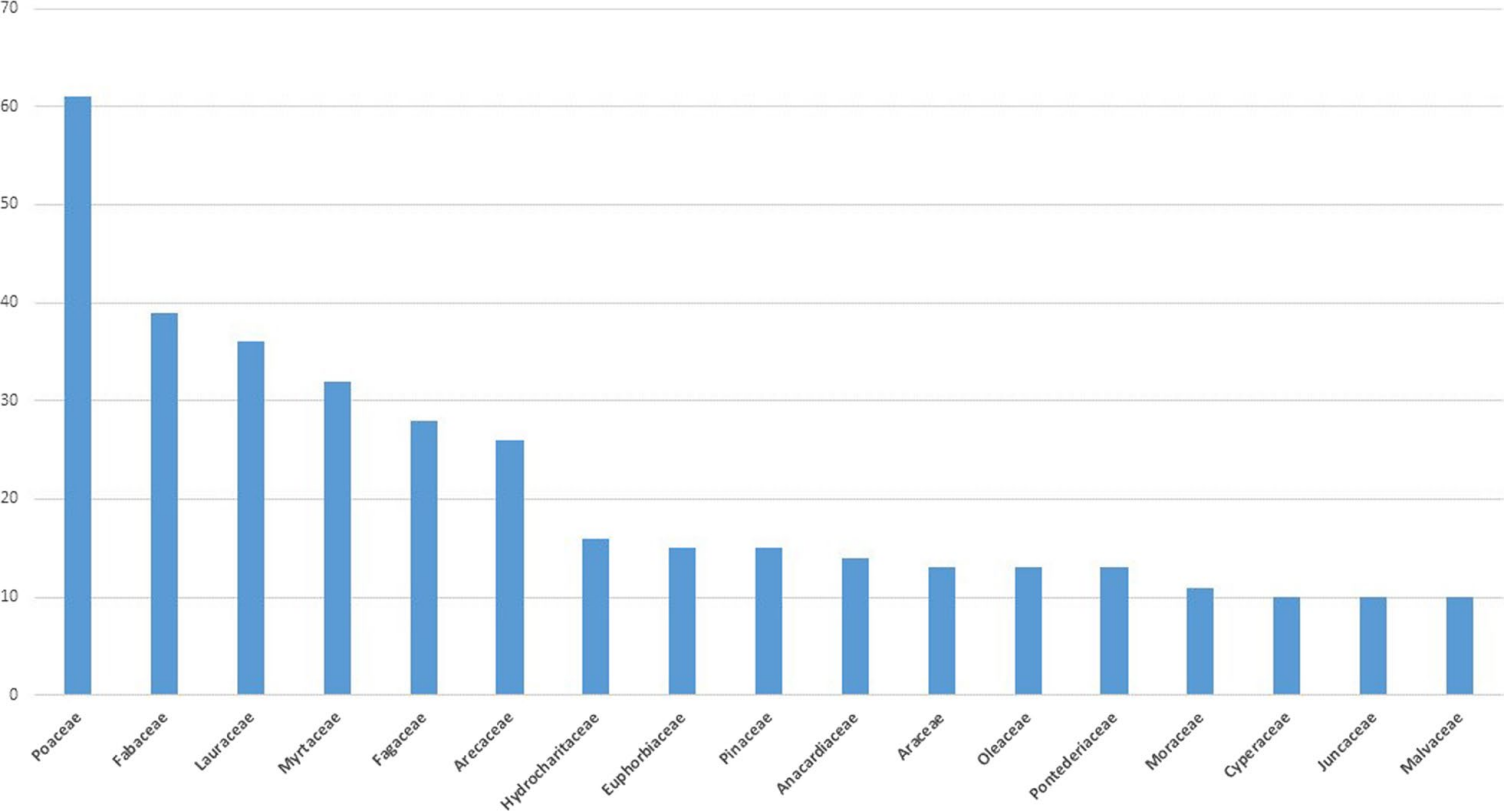
Bar graph of the most frequently reported families in all the reviewed studies, indicating the number of reports per family

Figure 9 shows the mostly reported families (minimum 10 occurrences) cited in the studies worldwide; the other 95 families had less than 10 occurrences each, which together constitute more than half of the families reported (Table 2).

**Table 2:** List of most diverse families reported in reviewed articles. Below is the table of the families most diversely used with more than eight uses

Family	Total use reports	Use	Taxa	Region covered	References
Anacardiaceae	14	Basketry	*Pistacia lentiscus*	Italy	[1]
		Catching fish	*Mangifera indica*	Brazil	[41]
			*Spondias mombin*	Brazil	[41]
			*Tapirira guianensis*	Brazil	[35]
		Dye nets	*Pistacia lentiscus*	Italy	[1]
			*Rhus coriaria*	Italy	[28]
		Fish poison	*Rhus coriaria*	Italy	[28]
		Fish traps	*Pistacia lentiscus*	Italy	[1]
		Fishing gear	*Anacardium occidentale*	Brazil	[42]
		Hulls	*Gluta tourtour*	Madagascar	[43]
		Illegal fishing	*Pistacia lentiscus*	Italy	[1]
		Splash battress	*Gluta tourtour*	Madagascar	[43]
		Fishing	*Schinus terebinthifolia*	Brazil	[31]
Arecaceae	25	Broom heads	*Attalea funifera*	Brazil	[30]
		Catching fish	*Acrocomia aculeata*	Brazil	[41]
			*Attalea funifera*	Brazil	[30]
			*Attalea phalerata*	Brazil	[41]
			*Bactris glaucescens*	Brazil	[41]
			Bactris spp.	Brazil	[41]
			*Copernicia alba*	Brazil	[41]
			*Euterpe edulis*	Brazil	[35]
			*Geonoma schottiana*	Brazil	[35]
			*Syagrus romanzoffiana*	Brazil	[35]
		Control of snails	*Cocos nucifera*	India	[44]
			*Phoenix dactylifera*	India	[44]
		Fish nets	*Chamaerops humilis*	Italy	[1]
			*Cocos nucifera*	Italy	[1]
		Fishing	*Chamaerops humilis*	Italy	[1]
		Fishing gear	*Copernicia prunifera*	Brazil	[42]
		Food for fish	*Astrocaryum jauari*	Brazil	[7]
			*Euterpe precatoria*	Brazil	[8]
		Hiding place	*Euterpe precatoria*	Brazil	[8]
		Ropes	*Chamaerops humilis*	Italy	[1]
			*Cocos nucifera*	Italy	[1]
		Shrouds	*Chamaerops humilis*	Italy	[1]
		Stabilize temperature	*Euterpe precatoria*	Brazil	[8]
		Catching fish	*Astrocaryum jauari*	Suriname	[45]
Euphorbiaceae	15	Bait	*Euphorbia dendroides*	Italy	[28]
		Catching fish	*Euphorbia dendroides*	Italy	[28]
			*Sapium obovatum*	Brazil	[41]
		Fish poison	*Jatropha curcas*	India	[46]
		Fishing	*Euphorbia characias*	Italy	[1]
		Food for fish	*Hevea brasiliensis*	Brazil	[7]
			*Mabea subsessilis*	Brazil	[7]
		Hulls	*Givotia madagascariensis*	Madagascar	[43]
		Illegal fishing	*Euphorbia characias*	Italy	[47]
			*Euphorbia dendroides*	Italy	[1]
			*Euphorbia helioscopia*	Italy	[1]
		Making fishing rods	*Sebastiania schottiana*	Brazil	[31]
		Signify sharks giving birth	*Excoecaria agallocha*	Fiji	[48]
Fabaceae	38	Bait	*Copaifera guianensis*	Suriname	[45]
			*Dioclea guianensis*	Suriname	[45]
			*Macropsychanthus scaber*	Suriname	[45]
		Build boats	*Apuleia leiocarpa*	Brazil	[31]
			*Enterolobium contortisiliquum*	Brazil	[31]
			*Myrocarpus frondosus*	Brazil	[31]
			*Parapiptadenia rigida*	Brazil	[31]
		Build canoes	*Schizolobium parahyba*	Brazil	[49]
		Build ships	*Ceratonia siliqua*	Italy	[1]
			*Laburnum anagyroides*	Italy	[1]
			*Robinia pseudoacacia*	Italy	[1]
		Catching fish	*Balizia pedicellaris*	Brazil	[35]
			*Inga vera*	Brazil	[41]
			*Ormosia arborea*	Brazil	[35]
			*Tamarindus indica*	Philippines	[50]
		Control of unwanted fishes	*Gliricidia sepium*	India	[44]
		Faster in hatching	*Acacia* sp.	India	[44]
		Fish conservation	*Pithecellobium dulce*	Philippines	[50]
		Fish poison	*Albizia odoratissima*	India	[51]
			*Millettia pachycarpa*	India	[46]
			*Tephrosia sinapou*	Suriname	[45]
		Fishing	*Inga virescens*	Brazil	[31]
		Fishing gear	*Mimosa caesalpiniifolia*	Brazil	[42]
		Fishing net floats	*Erythrina crista-galli*	Brazil	[31]
		Food for fish	*Inga disticha*	Suriname	[45]
		Hooks	*Acacia karroo*	Italy	[1]
		Hulls	*Entada pervillei*	Madagascar	[43]
		Repair canoes	*Schizolobium parahyba*	Brazil	[49]
		Signify octopus season	*Erythrina variegata*	Fiji	[48]
		Splash battress	*Entada pervillei*	Madagascar	[43]
		Stabilize soil	*Inga edulis*	Peru	[52]
			*Swartzia simplex*	Peru	[52]
Fagaceae	24	Build boats	*Apuleia leiocarpa*	Brazil	[31]
		Barrels	*Castanea sativa*	Italy	[1]
			*Fagus sylvatica*	Italy	[1]
			*Quercus pubescens*	Italy	[1]
		Basketry	*Castanea sativa*	Italy	[1]
			*Quercus suber*	Italy	[1]
		Build boats	*Quercus cerris*	Italy	[47]
			*Quercus pubescens*	Italy	[47]
		Build ships	*Castanea sativa*	Italy	[1]
			*Fagus sylvatica*	Italy	[1]
			*Quercus pubescens*	Italy	[1]
			*Quercus robur*	Italy	[1]
			*Quercus* sp.	Italy	[1]
		Dye nets	*Castanea sativa*	Italy	[1]
		Fish nets	*Castanea sativa*	Italy	[1]
		Fish traps	*Castanea sativa*	Italy	[1]
		Floats	*Quercus suber*	Italy	[47]
		Grilling	*Castanea sativa*	Italy	[1]
		Make oars	*Castanea sativa*	Italy	[47]
		Mussel farming	*Castanea sativa*	Italy	[1]
		Ramps	*Quercus pubescens*	Italy	[1]
		Tools	*Castanea sativa*	Italy	[1]
			*Fagus sylvatica*	Italy	[1]
		Weaving fish traps	*Quercus pubescens*	Italy	[47]
		Barrels	*Quercus robur*	Italy	[1]
		Build ships	*Quercus ilex*	Italy	[1]
		Mussel farming	*Quercus ilex*	Italy	[1]
		Ramps	*Quercus ilex*	Italy	[1]
Hydrocharitaceae	16	Catching fish	*Hydrilla verticillata*	India	[53]
			*Hydrilla verticillata*	Philippines	[50]
			*Hydrocharis morsus-ranae*	Hungary	[54]
		Fish habitat	*Enhalus acoroides*	Indonesia	[55]
			*Hydrilla verticillata*	Philippines	[56]
			*Thalassia hemprichii*	Kiribati	[57]
			*Vallisneria natans*	Philippines	[50, 56]
		Food for fish	*Hydrilla verticillata*	India	[9]
			*Hydrocharis spongia*	Brazil	[8]
			*Thalassia hemprichii*	Kiribati	[57]
		Food for people	*Enhalus acoroides*	Indonesia	[55]
		Hiding place	*Hydrocharis spongia*	Brazil	[8]
		Problem for fishing activities transportation	*Egeria densa*	Kenya	[58]
		Source of income	*Enhalus acoroides*	Indonesia	[55]
		Stabilize temperature	*Hydrocharis spongia*	Brazil	[8]
Malvaceae	10	Bait	*Pachira insignis*	Suriname	[45]
		Build canoes	*Ceiba pentandra*	Madagascar	[43]
		Catching fish	*Pseudobombax marginatum*	Brazil	[41]
		Caulking	*Gossypium* spp.	Italy	[1]
		Fish nets	*Gossypium* spp.	Italy	[1]
		Fish traps	*Gossypium* spp.	Italy	[1]
		Fishing	*Gossypium* spp.	Italy	[1]
		Food for fish	*Pseudobombax munguba*	Brazil	[8]
		Hiding place	*Pseudobombax munguba*	Brazil	[8]
		Stabilize temperature	*Pseudobombax munguba*	Brazil	[8]
Myrtaceae	32	Bait	*Myrtus communis*	Italy	[1]
		Basketry	*Myrtus communis*	Italy	[1]
		Build boats	*Eucalyptus* sp.	Brazil	[31]
		Build temporary fishing camps	*Eucalyptus* sp.	Brazil	[31]
		Catching fish	*Eugenia astringens*	Brazil	[35]
			*Eugenia stigmatosa*	Brazil	[35]
			*Eugenia sulcata*	Brazil	[35]
			*Gomidesia fenzliana*	Brazil	[35]
			*Gomidesia schauerian*	Brazil	[35]
			*Myrcia bicarinata*	Brazil	[35]
			*Myrcia glabra*	Brazil	[35]
			*Myrcia glomerata*	Brazil	[35]
			*Myrcia hebepetala*	Brazil	[35]
			*Myrcia macrocarpa*	Brazil	[35]
			*Myrcia multiflora*	Brazil	[35]
			*Myrcia pubipetala*	Brazil	[35]
			*Myrcia racemosa*	Brazil	[35]
			*Myrcia* sp.	Brazil	[35]
			*Myrcia splendens*	Brazil	[35]
			*Myrcia vellozoi*	Brazil	[35]
			*Neomitranthes glomerata*	Brazil	[35]
			*Pimenta pseudocaryophyllus*	Brazil	[35]
			*Psidium cattleyanum*	Brazil	[35]
			*Psidium guajava*	Brazil	[41]
			*Psidium guineense*	Brazil	[41]
			*Siphoneugena guilfoyleiana*	Brazil	[35]
		Fish traps	*Myrtus communis*	Italy	[1]
		Repair fishing nets	*Myrtus communis*	Italy	[28]
		Tools	*Myrtus communis*	Italy	[1]
Oleaceae	13	Barrels	*Fraxinus ornus*	Italy	[1]
		Basketry	*Olea europaea*	Italy	[1]
		Build ships	*Fraxinus ornus*	Italy	[1]
			*Olea europaea*	Italy	[1]
		Catching fish	*Chionanthus filiformis*	Brazil	[35]
		Fish traps	*Fraxinus ornus*	Italy	[1]
			*Olea europaea*	Italy	[1]
		Fishing	*Olea europaea*	Italy	[1]
		Ramps	*Olea europaea*	Italy	[1]
		Tools	*Fraxinus ornus*	Italy	[1]
			*Olea europaea*	Italy	[1]
			*Phillyrea angustifolia*	Italy	[1]
Poaceae	53	Aids in fishery management		India	[27]
		Basketry	*Ampelodesmos mauritanicus*	Italy	[1]
			*Arundo plinii*	Italy	[1]
		Build ships	*Arundo donax*	Italy	[1]
		Catching fish	*Arundo donax*	India	[9]
		Catching fish	*Arundo donax*	Italy	[47]
			*Bambusa* sp.	India	[51]
			*Glyceria maxima*	Hungary	[54]
			*Hygroryza* sp.	India	[53]
				India	[51]
			*Phragmites australis*	Hungary	[54]
		Control of humus gas	*Bambusa* sp.	India	[44]
		Control of snails	*Bambusa* sp.	India	[44]
		Dams of fishponds	*Arundo donax*	Italy	[1]
			*Phragmites australis*	Italy	[1]
		Fish habitat	*Leersia hexandra*	India	[53]
			*Phragmites karka*	India	[53]
			*Tripidium bengalense*	India	[53]
		Fish nets	*Arundo donax*	Italy	[1]
			*Lygeum spartum*	Italy	[1]
		Fish traps	*Arundo donax*	Italy	[1]
			*Arundo plinii*	Italy	[1]
			*Bambusa* sp.	India	[59]
			*Oryza* sp.	India	[59]
		Food for fish	*Hymenachne amplexicaulis*	Brazil	[8]
			*Oryza grandiglumis*	Brazil	[8]
			*Oryza* sp.	India	[44]
			*Paspalum repens*	Brazil	[8]
			*Phragmites australis*	Hungary	[54]
				India	[53]
			*Rugoloa polygonata*	Brazil	[8]
		Help lower the cost of fish feed	*Bambusa* sp.	India	[59]
		Hiding place	*Hymenachne amplexicaulis*	Brazil	[8]
			*Oryza grandiglumis*	Brazil	[8]
			*Paspalum repens*	Brazil	[8]
			*Rugoloa polygonata*	Brazil	[8]
		Making fish traps	*Phragmites*	Kenya	[58]
		Prevent mortality in transportation	*Oryza* sp.	India	[44]
		Protects fish from predators	*Bambusa* sp.	India	[59]
		Ropes	*Lygeum spartum*	Italy	[1]
		Stabilize temperature	*Hymenachne amplexicaulis*	Brazil	[8]
			*Oryza grandiglumis*	Brazil	[8]
			*Paspalum repens*	Brazil	[8]
			*Rugoloa polygonata*	Brazil	[8]
		Tools	*Arundo donax*	Italy	[1]
		Basketry	*Ampelodesmos mauritanicus*	Italy	[28]
			*Arundo donax*	Italy	[1]
		Fish nets	*Ampelodesmos mauritanicus*	Italy	[28]
		Fish nets	*Ampelodesmos mauritanicus*	Italy	([1])
		Fish traps	*Ampelodesmos mauritanicus*	Italy	[1]
		Mussel farming	*Ampelodesmos mauritanicus*	Italy	[1]
		Ropes	*Ampelodesmos mauritanicus*	Italy	[28]
		Ropes	*Ampelodesmos mauritanicus*	Italy	[1]
Pontederiaceae	13	Control algal bloom	*Pontederia crassipes*	India	[44]
		Fish habitat	*Pontederia crassipes*	India	[53]
		Fish habitat	*Pontederia crassipes*	Philippines	[56]
		Food for fish	*Pontederia crassipes*	Brazil	[8]
			*Pontederia rotundifolia*	Brazil	[8]
		For harvesting fish	*Pontederia crassipes*	India	[51]
		Hiding place	*Pontederia crassipes*	Brazil	[8]
			*Pontederia rotundifolia*	Brazil	[8]
		Invasive, causing increase in *Pistia stratiotes*, *Azolla pinnata*, and *Trapa natans*	*Pontederia crassipes*	Kenya	[58]
		Problem contributing to fish kill	*Pontederia crassipes*	Philippines	[56]
		Problem for fishing activities transportation	*Pontederia crassipes*	Philippines	[56]
		Stabilize temperature	*Pontederia crassipes*	Brazil	[8]
			*Pontederia rotundifolia*	Brazil	[8]

**Fig. 10:**
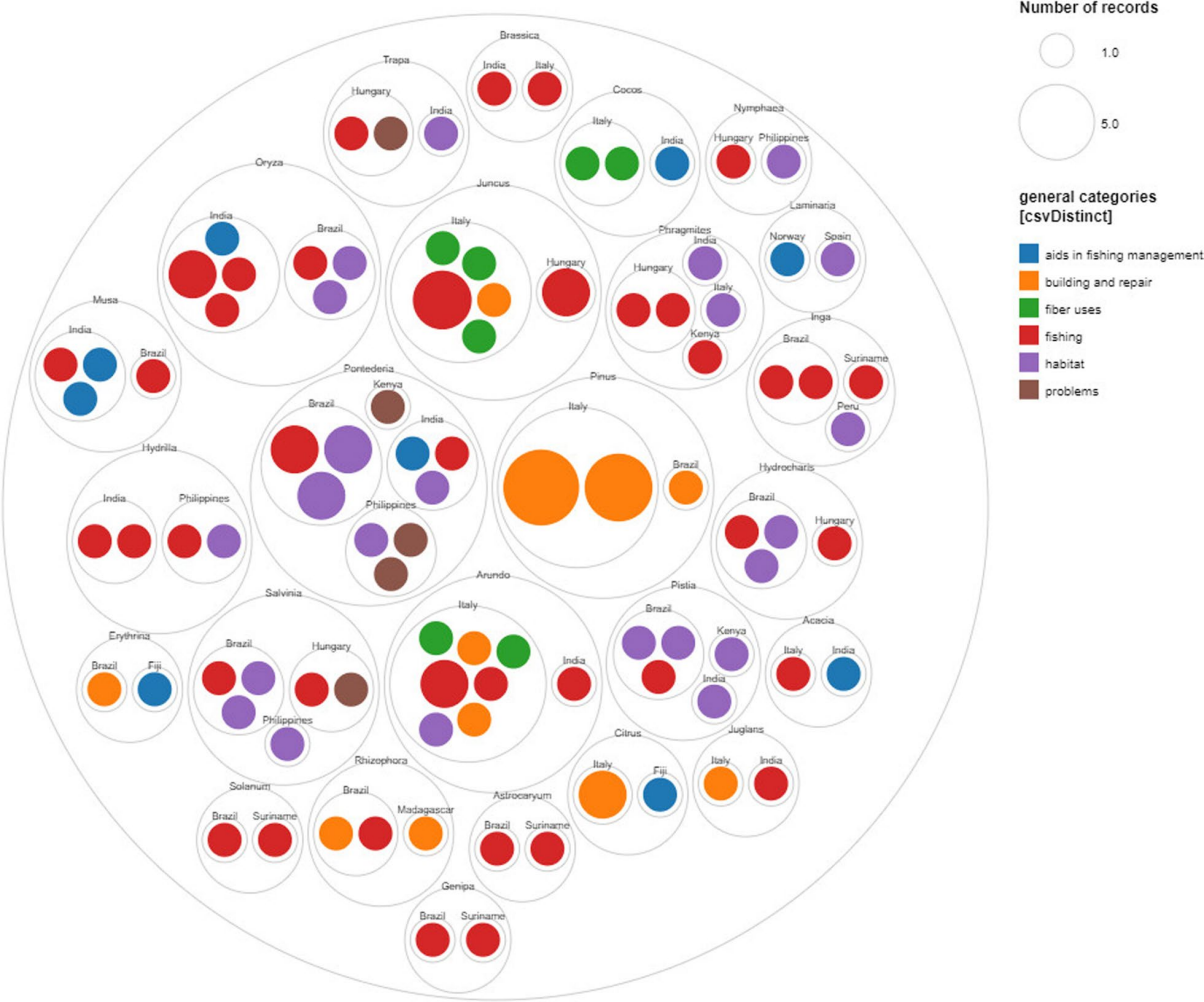
Shared species used within genera across countries

A total of 41 uses involving 25 genera are shared within different localities, while the remaining uses are utilized in a single region. Figure 10 illustrates the use of similar genera for similar purposes across countries. Within-family diversity can be observed, but there is also species overlap; therefore, some species are used similarly across countries.

### Fishing-related uses

There is a high variety in the fishing general category; however, most of the uses have been recorded in Brazil. Among the most reported families for this category are Myrtaceae, Poaceae, Arecaceae, and Fabaceae. Specifically, the number of families recorded in each use are as follows: catching fish (57), food for fish (36), fish poison (16), fish traps (12), fishing (11), bait (7), illegal fishing (4), fishing gear (5), harvesting fish (1), hooks (1), making fish traps (1), making fishing rods (1), mussel farming (2), to get octopus out of its den (1), to stun fishes (1). With regard to these fishing-related uses, the following taxa are shared among fishers from India, Italy, Brazil, Suriname, the Philippines, Hungary, and Kenya: *Acacia*, *Arundo*, *Astrocaryum*, *Brassica*, *Genipa*, *Hydrilla*, *Hydrocharis*, *Inga*, *Juglans*, *Juncus*, *Musa*, *Nymphaea*, *Oryza*, *Phragmites*, *Pontederia*, *Rhizophora*, *Salvinia Solanum*, and *Trapa* are shared among India, Italy, Brazil, Suriname, the Philippines, Hungary, and Kenya. Specifically, *Arundo donax* is used in India for catching fish [9] and in Italy as well [47]. The taxon *Astrocaryum jauari* is used in Brazil as food for fish [7] and in Suriname for catching fish [45]. *Brassica juncea* is used in India as food for fish [44], while *Brassica oleracea* is used in Italy as bait [1]. *Genipa americana* is used for catching fish in Brazil [41] and as bait in Suriname [45]. *Hydrilla verticillata* is used as food for fish in India [9] and for catching fish in the Philippines and India [50, 53]. *Hydrocharis spongia* is used as food for fish in Brazil [8], while in Hungary *Hydrocharis morsus-ranae* is used for catching fish [54]. In Brazil, *Inga vera* is used for catching fish [41], while *Inga virescens* is used for fishing [31]. However, in Suriname, *Inga disticha* is used as food for fish [45]. In Hungary, both *Juncus bufonius* and *Juncus effuses* are used for recreational fishing [54]. In Italy, *Juncus acutus*, *Juncus spp*., and *Juncus maritimus* are used in making fish traps [1]. In India, *Musa sp*. is used for catching fish [51], while in Brazil, specifically, *Musa acuminata* is used for fishing gear [42]. In India, *Oryza sp*. is used for catching fish [51] and fish traps [59], while *Oryza grandiglumis* is used as food for fish in Brazil, while *Oryza sativa* and more generally *Oryza sp.* in India [8, 44, 53]. *Phragmites australis* is used for catching fish and as food for fish in Hungary [54], while in Kenya *Phragmites* is generally used for making fish traps [58]. In Brazil, the taxa *Pontederia crassipes* and *Pontederia rotundifolia* are used as food for fish [8], while in India *Pontederia crassipes* is used for harvesting fish [51]. *Salvinia minima* is also used in Brazil as food for fish [8], while *Salvinia natans* is used in Hungary for catching fish [54]. *Solanum viarum* is used for catching fish in Brazil [41], while *Solanum schomburgkii* is used as food for fish in Suriname [45].

### Building and repair-related uses

Most building and repair uses are reported in Italy, followed by Brazil and Madagascar, and were not reported in other regions. Among the most reported families are Lauraceae, Fagaceae, Fabaceae, and Pinaceae. For this category, the number of families recorded in each use are as follows: build ships (16 citations), build boats (10), tools (7), build canoes (19), dye nets (11), ramps and repair canoes (5 each), barrels and hull (3 each), and build temporary fishing camps, caulking, splash bmattress, repair fishing nets, fishing net floats, and fuel for cooking fish (2 each). The remaining uses are represented by only one family: coloring, constructing boat shelters, covering boats and floats, grilling, make oars, make shifts go faster, masts, paddles, pulleys, ship models, and shrouds.

The use of the genera *Pinus* and *Rhizophora* is shared among Brazil, Italy, and Madagascar. *Pinus* spp. is used in Brazil to build boats [31], while in Italy, it is used in building ships and dye nets [1, 47]. The taxon *Rhizophora mangle* is used in Brazil to construct boat shelters [30], while in Madagascar, *Rhizophora sp.* is used for masts [43].

For building boats in Brazil, for instance, Baptista et al. [31] mentioned that the “timbaúva” (*Enterolobium contortisiliquum*) and “cedro” (*Cedrela fissilis*) were utilized for boat building, and Hanazaki [60] demonstrated that these species were also used by traditional fishers “caiçaras,” in southern Brazil. It appears that using plant species to make boats was an important activity in the past in many regions of Brazil, as mentioned by local fishers in the state of Alagoas, Northeast Brazil [61]. However, Baptista et al. [32] argued that this had been replaced of new materials, which has caused fishers to lose this knowledge given that, in the past, few fishers reported this plant use. The active of retired fishermen generally know more about wood- and fiber-producing plants [62] and specifically regarding using plants for construction, such as building boats and fishing artifacts [30].

### Fiber-related uses

Some studies provide information not present in other countries, such as fiber-related uses. Among the most reported families are Poaceae, Arecaceae, Cannabaceae, and Fagaceae. The number of families recorded in each use is as follows basketry (10), fish nets (7), ropes (6), weaving fish traps (2), while the remaining uses, such as broom heads and use as cordage in fishing activities are only represented by a single family. These uses related to fibers are reported most in a local Italian contexts, except for *Attalea funifera*, which is used to make broom heads in Brazil [30].

Habitat-related uses have been mainly reported in Brazil, followed by India, Fiji, and the Philippines, while Peru, Indonesia, Kiribati, Kenya, Spain, and Italy are among the least reported regions. For this category, among the most reported families are Poaceae, Araceae, Hydrocharitaceae, Pontederiaceae, and Cyperaceae. The number of families recorded for each use is as follows: fish habitat (22), stabilize temperature (19), hiding place (18), and stabilize soil (3), while the remaining uses, such as dams of fishponds, help lower the cost of fish feed, protect fish from predators, and provide shade are among the least represented. The use of *Phragmites*, *Pistia*, *Pontederia*, and *Salvinia*, for the above-mentioned purposes, is shared among Italy, India, Brazil, Kenya, and the Philippines considering dams of fish ponds, fish habitat, hiding place, and stabilize temperature [1, 8, 53, 56, 58]. Specifically, *Phragmites australis* is used in fishpond dams in Italy [1], while in India *Phragmites karka* is used as fish habitat [53]. *Pistia stratiotes* is used as a hiding place and to provide a stable temperature for fish in Brazil [8]. In Kenya and India, this taxon is used as a fish habitat [53, 58]. *Pontederia crassipes* is used as a hiding place and *Pontederia rotundifolia* to provide a stable temperature for fish in Brazil [8], while in the Philippines and India, the taxon *Pontederia crassipes* is used as a fish habitat [46, 56]. *Salvinia minima* is also used as a hiding place and to provide a stable temperature for fish in Brazil [8]. In addition, in the Philippines, the taxon *Salvinia molesta* is used as a fish habitat [56].

### Aids in fishing management

Most of the aids in fishing management uses have been reported in India, Fiji, Norway, Indonesia, and the Philippines and not in other regions. Among the most reported families are Fabaceae, Poaceae, and Musaceae. For this category, the number of families recorded in each use is as follows: water filter (10 citations), mapping of fish resources (4), control of humus gas, control of snails, signify octopus season, and the control of wild cat (2). The remaining uses are represented by only one family: control disease in fish, reduction of water turbidity, check pH, control algal bloom, control of unwanted fishes, faster in hatching, fish conservation, prevent mortality in transportation, seasonal cues for fish presence, signify sharks giving birth, and site for catching fish. Moreover, in India, the following taxa and uses are mentioned: *Acacia* sp. to allow the faster hatching of fish eggs, *Cocos nucifera* to control snails, *Musa* sp. to control of humus gas and the reduction of water turbidity, *Pontederia crassipes* to control algal bloom, and *Oryza* sp. to prevent fish mortality in transportation [44, 59]. In Fiji, however, *Citrus reticulata* is used as a seasonal cue for fish presence and *Erythrina variegate* is used to signify octopus season [48].

### Plants causing problems

The plants causing problems are the least mentioned category and have been reported only in some countries, such as Hungary, Indonesia, Kenya, and the Philippines. Although not yet reported in other countries, these plants could be crucial for the management of fisheries across the globe. Among the most reported families causing problems are Hydrocharitaceae and Pontederiaceae. There are four families causing problems for recreational fishing (Ceratophyllaceae, Lythraceae, Menyanthaceae, and Salviniaceae, Hungary [54]), two families contributing to fish kills (Microcystaceae and Pontederiaceae, Philippines [56]), and two families causing problems for fishing activities transportation (Hydrocharitaceae and Pontederiaceae, Philippines [56] and Kenya [58]).

The problems caused by the genus *Pontederia* are similar in Kenya and the Philippines. In Kenya, *Pontederia crassipes* is considered invasive as it is causing an increase in other species such as *Pistia stratiotes*, *Azolla pinnata*, and *Trapa natans* [58], while in the Philippines this species is considered to be causing problems for transportation related to fishing activities as well as contributing to fish kill events [56].

## Discussion

### The role of plants in fishery

Plants in fishery activities are chosen primarily for their characteristics which make them suitable for specific environment and uses, as per Savo et al. [1]; for example, if a species is woody or is located in riverine areas, it may serve as fish habitat. Additionally, various factors influence the selection of fishing gear and techniques, including the following: the standard of living, properties of the raw material, nature of fish stock, and the physiography of the given water body [63]. For example, in Brazil, the traditional harvesting techniques, such as those used by “caiçaras,” are dependent on the desired characteristics needed to build a fixed fishing trap called “cerco-fixo” [35]. Similarly, when fishing with attractants (*Chali diya*), a bunch of *Eichhornia crassipes* are used to help identify the exact position of dough at the time of fishing with cast nets [51]. Also, Kalita et al. [51] reported additional unique indigenous knowledge related to fish harvesting, including fishing with piscicidal plants (*Polygonum hydropiper, Albizzia odoratissima, and Duranta plumieri*), community fishing (harvesting fish in groups), wounding gear (use of a weapon, e.g., a spear), and "bana" fishing (screen made from bamboo strips). The traditional fish aggregating device called “yankaw” in Laguna lake Philippines is made from branches of tamarind (*Tamarindus indica*) or kamatsili (*Pithecellobium dulce*) providing shelter and a protected area for fish, which is part of the fishery resource management [50]. Traditional fish traps could also include using *Arundo donax* [9], *Bambusa* sp., and *Oryza* sp. [59]. However, fish traps are currently made from plastic or metal materials which can be easily folded and that last longer, according to the local people in Santa Marinella and Civitavecchia, but which are considered less effective according to the Amalfi Coast informants in Western Mediterranean coast of Italy [1].

The reviewed publications reported new biological information on various plant species which serve as food items for fish, showing their dependency on forests as food sources. For instance, fruits are one of the most cited foods for fish by local fishers, aside from other fish, terrestrial invertebrates, and detritus. In line with these observations, important ecological processes concerning energetic pathways and food webs could be inferred with respect to the clear and blackwater rivers of the Amazonian floodplain; this shows the ecological link between ethnobiological research on LEK to the protection of Brazilian streams, which was not recorded in the previous literature [7]. The fish could be dispersers of aquatic macrophytes and riparian tree seeds to upstream germination areas [64]. This could be because fish gather most of their energy from fruits apart from insects, from flooded forests and terrestrial habitats due to the low productivity in the Negro River [7, 65]. Knöppel [66] argued that the fact that fish eat plants that occur in several habitat types in Central Amazonian streams could possibly be an adaptation. Further studies on this feeding interaction could allow the discovery of new information. Furthermore, Silvano et al. [7] demonstrated that their findings helped understand how deforestation impacts could affect fisheries and how declining fish populations could negatively affect ecological processes, including seed dispersal in terrestrial areas. Additionally, Silvano et al. [7] mentioned this could be crucial information beneficial to designing ecosystem management measures. This could further advance the management of the artisanal fisheries in the Amazon with the engagement of fishers [67, 68]. Previous studies proved that knowledge of local fishers regarding fish ecology and behavior has the potential to guide the sustainable management of the Amazon region, including flooded forests resulting from the impacts of climate change and other activities such as deforestation and mining [7, 45]. Likewise, other food sources for fish include seagrass, such as *Thalassia hemprichii* [57] and cyanobacteria *Microcystis aeruginosa* [56], and thus, the decline of these food sources may result to fish starvation and could affect their size.

Moreover, floating vegetation islands such as the Amazonian “matupás” which act as hiding places and provide fishes with stable temperatures (also see Silvano et al. [15] for microclimate provision) are found in floodplain lakes of the central Brazilian Amazon that started from the agglomeration of aquatic vegetation which then accumulated enough organic matter to grow patches of forest up to 12 m in height and to an area of several hectares, thus also contributing indirectly to fish abundance [8]. Tannins and flavonoids from branches of trees used as traditional fishing gear, e.g., “yankaw,” improve the quality of water favorable for aquatic plants such as (*Hydrilla verticillata*), creating a suitable area for the reproduction of fishes [69]. At the same time, the roots of *Pistia stratiotes* are used to attach fertilized eggs during fish propagation [58].

However, *Pontederia crassipes* is considered invasive, causing an increase in other plant taxa [58], and its overgrowth has some negative impacts on transportation and movement when a large area is covered by them (as also seen with taxon *Egeria dens*, see Sayer et al. [58]). The overgrowth of *Pontederia crassipes* and that of “liya” or *Microcystis aeruginosa*, together with other factors, such as shallow water depth, a decrease in oxygen in the region, solid waste, and polluted water from agriculture activities and chemical substances from hydropower plants, contribute to fish kill incidents according to local fishers [56].

Trees and bushes are used for building-related activities due to the fact they are easy to stock up on in the Mediterranean [1]; however, all the species of the temperate belt are used probably because the wood of the trees found in this habitat has features that are conducive for shipbuilding [70]. LEK on the use of plants for construction, like making tools or building boats, is not very well documented but is very crucial for traditional communities that mainly depend on fishing [35, 71]. For instance, the wood of many tree species is used by shipwrights in the Western Mediterranean coastal regions of Italy, much more so compared to other parts of the world (e.g., [70, 72–74]). Some species have been utilized for building the hulls of ships since the time of the Roman Empire [75]. Some specific uses are associated with regional practices, such as the traditional fish preservation method of using salt, which local people in Sicily, Italy, mainly utilize; however, it could also be that the data from the region were limited [1]. Then, the shared uses within Italy could be a result of past historical relationships and reciprocal commercial activities [1].

Fiber-related uses are mostly reported in Italy. The Sicilian ethnobotanical literature reports that similar uses of plants can be found more frequently in other small islands with similar economies [29, 76] and nearby areas [77]. For example, baskets made of fibers of chest nut trees are used for carrying fish are shared in Liguria (locally “cofone” or “cofuìn”) and Campania (“coffe”), and this could be due to their shared cultural background given the ancient cultural connections among these regions [78]. However, in the Pacific region, it is maintained that different plant names imply the long-term presence of plants in the region, while having similar product names and technologies indicates the more recent arrival and they are shared among local users on different islands, highlighting the complete differences in the historical and cultural backgrounds between Mediterranean and Pacific countries [1].

Understanding the perception of local fishers about freshwater ecosystems could enhance our understanding of the cultural uses of wood and fiber of plant species (e.g., [1, 78]), thus providing information that could not only fill the gaps in ecology [79], biodiversity monitoring [80], but also serve as guidance toward local management of habitats [11, 81, 82] as a tool for habitat restoration [83, 84], and sustainable management of local freshwater bodies [12, 85, 86]. Including local knowledge in the discussion is crucial in conserving the natural environments in which these people live [31, 35].

### The rapid decline of the knowledge

There is a lack of ethnobotanical studies related to fisheries in spite of the rapid erosion of traditional knowledge and practices dependent on plant diversity [72]. The artisanal fisheries are understudied and decreasing along the Western Mediterranean coast of Italy, which, based on the perceptions of local people, is due to the overabundance of fishing vessels within the 20-mile limit off the coast (see also [87]), legal restrictions, the decline in fish stocks (see, e.g., [88, 89]), changes in fish species, changes in the climate such as different seasonality, dolphin predation, and increasing costs [1]. Globally, these events are happening at an unprecedented rate [90, 91]. Other contributing factors include increased fuel and equipment costs and decreased human power [54, 92] as well as urbanization of coastal areas [93]. In addition, societal development factors play a part, such as operating tourism activities in places where communities live without considering the local culture and perception [94, 95]. On the other hand, La Rosa et al. [28] argued that many ethnobotanical uses of plants are lost from the popular tradition because of tourism changes in the local economy. Tng et al. [30] found that urbanization, land-use change, impacts on productive practices, and growth of other activities, including tourism, threaten the perception of cultural rural communities across the globe. Handicrafts, practices, and strategies by local people are in rapid decline as most ethnobotanical uses have been abandoned, which makes it more challenging to assess if a particular use is specific to a region or if it has already lost in other areas. There is cultural erosion of knowledge on traditional instruments, ships, tools, fishing-related practices because plant fibers and wood for making fishing equipment, including boats, have been replaced by new and cheaper materials available in the market. Another example is the use of plastic nets, which could become a significant source of marine plastic pollution [96, 97]. In addition, these uses, which are rapidly disappearing in the Mediterranean area, have maximum heritage value; therefore, small islands are crucial in this context as biocultural refugia to preserve the uses and practices of the local people [1, 29]. Likewise, deforestation and the loss of plant species in terrestrial areas could affect fisheries, while the decline in fish stocks poses a risk to the important environmental process such as seed dispersion [7]. Thus, the loss of forested areas and of areas with native vegetation, where plant raw materials are collected, together with the decline in traditional fisheries, can contribute to the loss of plant knowledge.

Considering that traditional fishing lifestyle is declining and the opportunity to explore further their diverse knowledge is decreasing every year, interviewing resource users such as recreational anglers is vital given their potential contributions to providing crucial data about plants in the freshwater bodies [54]. Most of the traditional knowledge remains in the memories of older community members who have maintained strong attachments to the traditional practices they mainly depend on for a living. These practices are in danger of being lost quickly [1, 29], and this threatens the role of the small Mediterranean islands communities as biocultural refugia [3] especially in transmitting local perception and practices [98]. The perceptions and concerns of stakeholders and resource users can greatly enhance ecosystem management strategies [99]. As long as management approaches reflect local communities’ LEK, ethnobiological surveys could aid local communities’ conservation efforts [6]. For instance, the findings that plants species are important for local food security and the quality of the ecosystem in the region is affected by deforestation [52] combined with scientific research, could result in conservation agreements and the creation of measures to monitor the reappearance of fish species as part of the management of fisheries and riparian forests from a social-ecological approach. This therefore demonstrates that ethnobiological studies may enhance communication between locals and scientists, bridging the gap between biological sciences and LEK [7]. Documenting LEK has the potential to contribute to different areas of ecology, including conservation biology and habitat management. Therefore, to maintain the resilience of socioecological systems and cultural diversity conservation, it is important to explore further patterns of this cultural knowledge present in artisanal fishing community [30].

## Conclusion

The fishery-related uses of plants are greatly under-documented across the world. In the reviewed articles, semi-structured interviews are most commonly used with local people, especially fishery experts, in investigating plant use knowledge within traditional fishing communities. At the same time, a small percentage of the publications are also based on secondary sources and reviews of ethnobotanical literature involving the participation of other local people and institutions. As a novelty, we have proposed a categorization of fishery-related uses. Fishing, the building/repair of fishing tools, and habitat-related uses are reported the most in the reviewed articles, while the records of plants related to fiber uses, providing aid in fishing management, and species causing problems are among the least mentioned; however, the latter are crucial in understanding the ecosystem of a region. Several taxa are used in the same etic, or even emic, domains in different parts of the world, especially on the genus level. For example, *Pontederia* and *Juncus* use reports are the most commonly shared in terms of records concerning aids in fishing management, habitat, fishing, and problems, while *Pinus* is mostly used for building and repair-related purposes in countries such as Hungary, Italy, Kenya, Brazil, India, and the Philippines. Several reviewed articles highlighted that this knowledge is declining rapidly as a result of socioenvironmental changes.

Our results show that the topic of fishery-related plants is important and rich if specifically targeted. It is also clear from this review that the subject is greatly understudied globally and in most parts of the world the information is of a casual and sporadic nature. Considering the rapid decline of knowledge highlighted in a large number of the articles, further systematic research on fishery-related uses of plants is needed, especially given its potential contribution to the sustainable management of fishery resources. Fishers are the primary group that can provide information on aquatic plants and algae that aid in fishing management. While the plants causing problems are the least reported globally, they are crucial in understanding the ecosystem of a region that is experiencing environmental challenges. As fishers can also describe in detail the plant species causing problems, we encourage the collaboration of scientists and fishers.

Therefore, considering the understudied nature of fishery-related uses of plants globally, further studies are needed to evaluate the plant-related knowledge of local resource users such as fisherfolk, given that they possess valuable ecological information vital for the sustainable management of local resources.

### Supplementary Information

**Additional file 1:** Review of plant species related to fisheries worldwide.

**Additional file 2:** List of Reviewed Articles related to fisheries found in Scopus and ISIWOS.

**Additional file 3:** List of Reviewed Articles related to fisheries NOT found in Scopus.

## Data Availability

All data used in this review can be found in the additional files.

## Abbreviations

TEK: Traditional ecological knowledge
LEK: Local ecological knowledge
PRISMA: Preferred Reporting Items for Systematic Reviews and Meta-Analyses
IUCN: International Union for Conservation of Nature
IUCN CEESP: IUCN Commission on Environmental, Economic and Social Policy
IUCN GSPFBU: IUCN Global Species Programme’s Freshwater Biodiversity Unit
UCSD: Uganda Coalition for Sustainable Development
NGOs: Non-government organizations
